# Combining Everolimus and Ku0063794 Promotes Apoptosis of Hepatocellular Carcinoma Cells via Reduced Autophagy Resulting from Diminished Expression of miR-4790-3p

**DOI:** 10.3390/ijms22062859

**Published:** 2021-03-11

**Authors:** Ho Joong Choi, Jung Hyun Park, Ok-Hee Kim, Kee-Hwan Kim, Ha Eun Hong, Haeyeon Seo, Say-June Kim

**Affiliations:** 1 Department of Surgery, Seoul St. Mary’s Hospital, College of Medicine, The Catholic University of Korea, Seoul 06591, Korea; ok6201@hanmail.net (O.-H.K.); hhe49@naver.com (H.E.H.); searcx12@naver.com (H.S.); sayjunekim@gmail.com (S.-J.K.); 2 Department of Surgery, Eunpyeong St. Mary’s Hospital, College of Medicine, The Catholic University of Korea, Seoul 03312, Korea; angle49@catholic.ac.kr; 3 Catholic Central Laboratory of Surgery, Institute of Biomedical Industry, College of Medicine, The Catholic University of Korea, Seoul 06591, Korea; keehwan@catholic.ac.kr; 4 Department of Surgery, Uijeongbu St. Mary’s Hospital, College of Medicine, The Catholic University of Korea, Seoul 11765, Korea

**Keywords:** autophagy, hepatocellular carcinoma, miR-4790-3p, mTOR inhibitor, ZNF225

## Abstract

It is challenging to overcome the low response rate of everolimus in the treatment of patients with hepatocellular carcinoma (HCC). To overcome this challenge, we combined everolimus with Ku0063794, the inhibitor of mTORC1 and mTORC2, to achieve higher anticancer effects. However, the precise mechanism for the synergistic effects is not clearly understood yet. To achieve this aim, the miRNAs were selected that showed the most significant variation in expression according to the mono- and combination therapy of everolimus and Ku0063794. Subsequently, the roles of specific miRNAs were determined in the processes of the treatment modalities. Compared to individual monotherapies, the combination therapy significantly reduced viability, increased apoptosis, and reduced autophagy in HepG2 cells. The combination therapy led to significantly lower expression of miR-4790-3p and higher expression of zinc finger protein225 (ZNF225)—the predicted target of miR-4790-3p. The functional study of miR-4790-3p and ZNF225 revealed that regarding autophagy, miR-4790-3p promoted it, while ZNF225 inhibited it. In addition, regarding apoptosis, miR-4790-3p inhibited it, while ZNF225 promoted it. It was also found that HCC tissues were characterized by higher expression of miR-4790-3p and lower expression of ZNF225; HCC tissues were also characterized by higher autophagic flux. We, thus, conclude that the potentiated anticancer effect of the everolimus and Ku0063794 combination therapy is strongly associated with reduced autophagy resulting from diminished expression of miR-4790-3p, as well as higher expression of ZNF225.

## 1. Introduction

Hepatocellular carcinoma (HCC) is the third leading global cause of death, with a 5-year survival rate of 10%. To date, sorafenib is the only regimen that is used to increase the survival rate of patients with advanced HCC. Everolimus is one of the inhibitors of the mammalian target of rapamycin (mTOR) and is designed for oral administration. In phase I/II trials, everolimus was shown to provide 3.8, 3.9, and 8.4 months of progression-free survival, time to progress, and overall survival for the patients with HCC, respectively [1]. Particularly, everolimus has shown its effectiveness to sorafenib-refractory patients [2]. Therefore, everolimus is expected to become a second-line therapy for sorafenib-refractory patients. However, the problem is that the response rate of everolimus to HCC is as low as 4%. To overcome this, many researchers have investigated methods to combine everolimus with other drugs.

Located downstream of phosphatidylinositol 3 kinase AKT pathways, mTOR plays a key role in the growth and proliferation of tumor cells. Furthermore, mTOR signaling is effective when it acts on the ribosomal protein S5 kinase beta-1 (S6K1) and eukaryotic entry factor-4-binding protein 1 (4E-BP1) [3]. Activated mTOR signaling is found in a variety of solid cancers, including HCC. In HCC, aberrant mTOR signaling is found in 48% of patients, which correlates with poor prognosis [4]. There are two complexes of mTOR—mTORC1 and mTORC2 [5,6]. Whereas mTORC1 regulates cell proliferation by phosphorylating S6K1 and 4E-BP1, mTORC2 does this by phosphorylating Akt. Theoretically, inhibiting mTORC1 alone does not completely inhibit tumor growth. When everolimus is used to suppress mTORC1, phosphorylation of S6K1 and 4E-BP1 is inhibited; however, tumor growth is possible via uninhibited mTORC2, which phosphorylates Akt [7]. Thus, an ideal mTOR inhibitor should inhibit both mTORC1 and mTORC2.

While everolimus is an mTORC1 inhibitor, Ku0063794 is a substance that can inhibit both mTORC1 and mTORC2. However, for some reason, previous studies indicated that everolimus and Ku0063794 did not differ significantly in terms of their anticancer efficacies against HCC [7,8]. Moreover, it was also revealed that combining everolimus and Ku0063794 potentiated the anticancer effect against HCC by significantly reducing autophagy [7,8]. However, the reason why autophagy increases in everolimus and Ku0063794 monotherapies but decreases in the combination therapy was not clearly defined [7]. In this study, we intended to investigate the mechanism of autophagy reduction induced by this combination therapy in relation to microRNA (microRNA) alternations.

## 2. Results

### 2.1. Comparison of Expression of miRNAs in Single and Combination Therapies of Everolimus and Ku0063794

**Figure 1:**
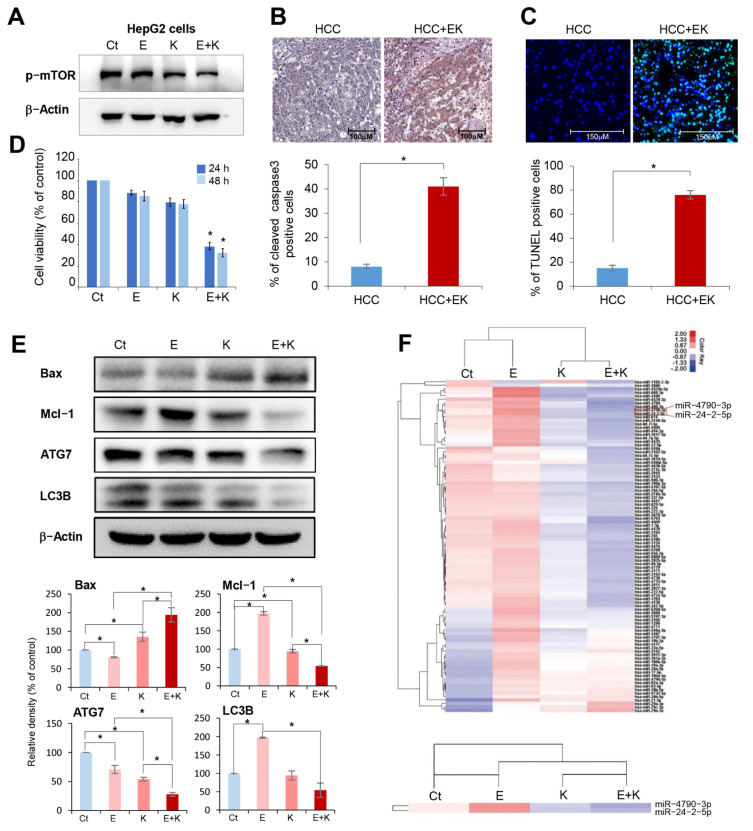
Comparison of expressions of miRNAs in mono- and combination therapies of everolimus and Ku0063794. (**A**) Western blot analysis showing the effects of each treatment on the expression of p-mTOR. (**B**) Cleaved caspase-3 immunohistochemistry with and without combination therapy in hepatocellular carcinoma (HCC) tissues. (**C**) TUNEL staining with and without combination therapy in HCC tissues. (**D**) Cell proliferation assay of HepG2 cells following mono- and combination therapies of 100 nM everolimus and 1 μM Ku0063794. Compared to the monotherapies of everolimus and Ku0063794, the combination therapy significantly reduced the viability of HepG2 cells. (**E**) Western blot analysis of HepG2 cells following mono- and combination therapies. Combination therapy induced a significant increase in the expression of Bax, a decrease in the expression of Mcl-1, and a decrease in the expression of autophagy markers. (**F**) The heat map of dysregulated miRNAs in HepG2 cells following mono- and combination therapies. Overall, everolimus monotherapy increased the expression of many miRNAs, Ku0063794 monotherapy reduced the expression of many miRNAs, and mixed therapies resulted in increased or decreased expressions of several miRNAs. Of all miRNAs, the alterations in the expression of miR-4790-3p and miR-24-2 were most prominent according to each treatment. Values are presented as means ± standard deviations of three independent experiments. Note: * *p* < 0.05. Abbreviations: Bax, Bcl-2-like protein 4; E, everolimus, K, Ku0063794, Mcl-1, myeloid cell leukemia 1.

We investigated the effects of everolimus and Ku0063794, alone or in combination, on cell apoptosis, proliferation, and autophagy in HepG2 cells. First, the effect of each treatment on the expression of p-mTOR was determined by Western blot analysis (Figure 1A). Combination therapy decreased the expression of p-mTOR compared to individual monotherapies. Next, leaved caspase-3 immunohistochemistry andterminal deoxynucleotidyl transferase dUTP nick end labeling (TUNEL)staining were performed to determine the effects of combination therapy on apoptosis of HCC tissues (Figure 1B,C). The combination therapy group exhibited a significantly higher numbers of cleaved caspase-3-positive and TUNEL-positive cells, respectively.

In addition, quantitative analysis of apoptosis using Annexin V staining and flow cytometry also validated the higher apoptotic cell proportion following combination therapy (Figure S1). In the cell viability assay, the combination therapy significantly reduced the viability of HepG2 cells compared to individual monotherapies (*p* < 0.05) (Figure 1D). Subsequently, Western blot analysis was performed to determine the alterations in cell apoptosis and autophagy when HepG2 cells were treated with everolimus or Ku0063794 alone or in combination (Figure 1E). The treatment with everolimus alone down- and upregulated the expression of Bax (an apoptotic marker) and Mcl-1 (an antiapoptotic marker), respectively; while Ku0063794 alone up- and downregulated them, respectively. The effects of individual monotherapies on the expression of autophagic markers (ATG7 and LC3B) were inconsistent. By contrast, combination therapy reduced the expression of autophagy markers (ATG7 and LC3B) and induced proapoptotic tendencies of apoptotic markers (higher expression of Bax and lower expression of Mcl-1) (*p* < 0.05). The autophagic flux (accumulation of LC3 expression in presence of autophagy inhibitor bafilomycin A1) also validated the reduced autophagy following combination therapy (Figure S2). Taken altogether, our results suggest that combination therapy significantly increases apoptosis of HepG2 cells and reduces autophagy compared to individual monotherapies. Similar results were also obtained in the experiments using Hep3B HCC cells (Figure S3, Supplementary Materials).

Next, we compared the expression of miRNAs in HepG2 cells following treatments with everolimus or Ku0063794 alone or in combination (Figure S4). Figure 1F illustrates the difference in the expression of miRNA in HepG2 cells following the single and combination therapies of everolimus and Ku0063794 using a heat map. Overall, everolimus monotherapy increased the expression of many miRNAs, Ku0063794 monotherapy reduced the expression of many miRNAs, and combination therapy resulted in increased or decreased expression of several miRNAs. Of all miRNAs, we found that alterations in the expression of miR-4790-3p and miR-24-2 were most prominent according to each treatment; the expression of these miRNAs was significantly increased following everolimus monotherapy, decreased following Ku0063794 monotherapy, and significantly decreased following combination therapy.

### 2.2. Changes in miR-4790-3p and miR-24-2-5p after Treatment with Everolimus or Ku0063794 Alone and in Combination

**Figure 2:**
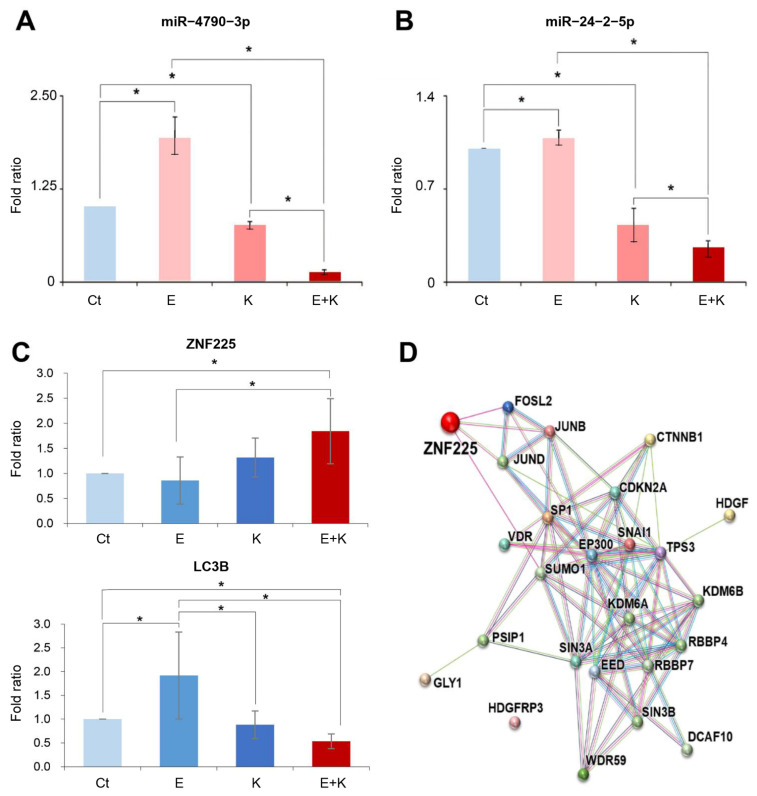
Changes in miR-4790-3p and miR-24-2-5p after everolimus and Ku0063794 mono- and combination therapies. (**A**,**B**) Real-time PCR showing the expression of miR-4790-3p and miR-24-2-5p in HepG2 cells after individual treatments. Compared to the control group, the expression of miR-4790-3p and miR-24-2-5p was increased after everolimus monotherapy, slightly decreased after Ku0063794 monotherapy, and significantly decreased after the combination therapy. (**C**) Real-time PCR showing the expression of Zinc finger protein 225 (ZNF225) and LC3B after the combination therapy. ZNF225 is known to be the target mRNA of miR-4790-3p. The mRNA expression of ZNF225 and LC3B was significantly up- and downregulated following combination therapy, respectively. (**D**) Potential network interactions of ZNF225 with JUNB, JUND, and FASL2 in the autophagy processes. ZNF225 is closely related with FOS like 2 (FOSL2), jun B proto-oncogene(JUNB), and jun D proto-oncogene(JUND), all of which play essential roles in the autophagic processes. The resulting network was computationally generated based on the databases (http://string-db.org. accessed date: 15 July 2019). The colors of the lines denote activation (green), inhibition (red), and unspecified interactions (gray). Values are presented as means ± standard deviations of three independent experiments. Note: * *p* < 0.05. Abbreviations: E, everolimus; K, Ku0063794.

We performed real-time PCR to detect the expression of miR-4790-3p and miR-24-2-5p in HepG2 cells after treating them with everolimus or Ku0063794 alone or in combination. Compared to the control group, the expression of miR-4790-3p was significantly increased after everolimus monotherapy, slightly decreased after Ku0063794 monotherapy, and significantly decreased after combination therapy (Figure 2A). The expression of miR-24-2-5p was similar to that of miR-4790-3p, but the range of the change was smaller (Figure 2B). Similar results were also obtained in the experiments using Hep3B HCC cells (Figure S5, Supplementary Materials).

The target of miR-4790-3p is predicted to be ZNF225 (http://www.targetscan.org. accessed date: 1 August 2015). ZNF225 is a protein that in humans is encoded by the ZNF225 gene. It belongs to the protein subtypes that contain the zinc finger, and little is known about its function. To determine its function, the mRNA expression of ZNF225 either with or without combination therapy was first investigated. The mRNA expression of ZNF225 was significantly increased in combination therapy compared to the control group (*p* < 0.05) (Figure 2C). The mRNA expression of ZNF225 was significantly increased in the group with combination therapy compared to the control and individual monotherapy groups (*p* < 0.05). The increased expression of ZNF225 following combination therapy could be attributed to the decreased expression of miR-4790-3p, which inhibits the expression of ZNF225. We also found that combination therapy significantly reduced the expression of LC3B, an autophagy marker, compared to monotherapy. Visualization of potential network interactions of ZNF225 revealed that ZNF225 is closely related to FOSL2, JUNB, and JUND, all of which play essential roles in the autophagic processes (Figure 2D).

### 2.3. Effects of miR-4790-3p and miR-24-2-5p on Autophagy in Combination Therapy

**Figure 3:**
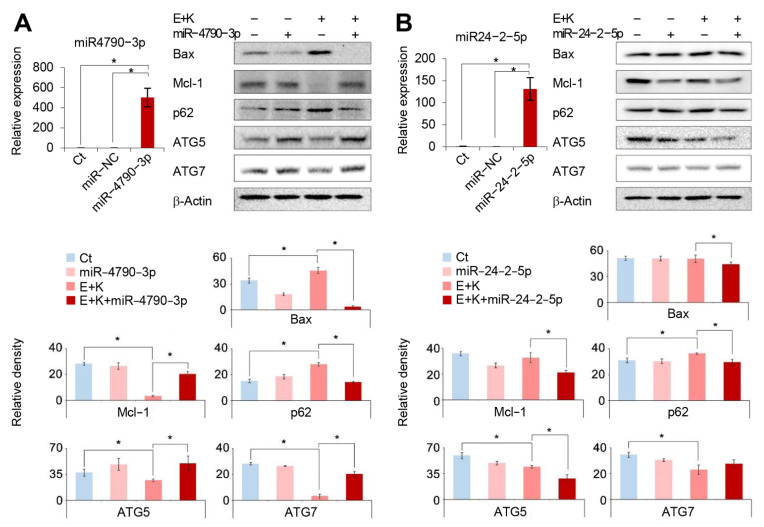
Overexpression test for the determination of the roles of miR-4790-3p and miR-24-2-5p. (**A**) (Left) Real-time PCR analysis demonstrating higher miR-4790-3p expression in miR-4790-3p-transfected HepG2 cells 24 h after transfection. (Right) Western blot analysis in HepG2 cells with miR-4790-3p overexpression following individual treatments. Overexpression of miR-4790-3p led to downregulation of Bax, upregulation of Mcl-2, and proapoptotic alterations (higher expression of ATG5 and ATG7 and lower expression of p62). (**B**) (Left) Real-time PCR analysis demonstrating higher miR-24-2-5p expression in miR-24-2-5p-transfected HepG2 cells 24 h after transfection. (Right) Western blot analysis in HepG2 cells with miR-24-2-5p overexpression following individual treatments. Overexpression of miR-24-2-5p did not lead to consistent alterations in the expression of proapoptotic, antiapoptotic, or autophagic proteins. Values are presented as means ± standard deviations of three independent experiments. Note: * *p* < 0.05. Abbreviations: Bax, Bcl-2-like protein 4; E, everolimus; K, Ku0063794; Mcl-1, myeloid cell leukemia 1; miR-NC, miRNA mimic negative control.

Next, we investigated the effects of miR-4790-3p and miR-24-2-5p on autophagy in HepG2 cells using a combination therapy. To achieve this aim, we performed Western blot analysis to determine the changes in apoptosis- and autophagy-related markers after overexpressing miR-4790-3p and miR-24-2-5p, respectively. When miR-4790-3p was overexpressed, we found that the apoptotic marker Bax decreased, the antiapoptotic marker Mcl-2 increased, and the altered autophagy-related factors (higher expression of ATG5 and ATG7 and lower expression of p62) suggested proautophagy (Figure 3A). In contrast, when miR-24-2-5p was overexpressed, there were no consistent changes in apoptosis- and autophagy-related factors, such as those observed during the overexpression of miR-4790-3p (Figure 3B). Similar results were also obtained in the experiments using Hep3B HCC cells (Figure S6, Supplementary Materials).

**Figure 4:**
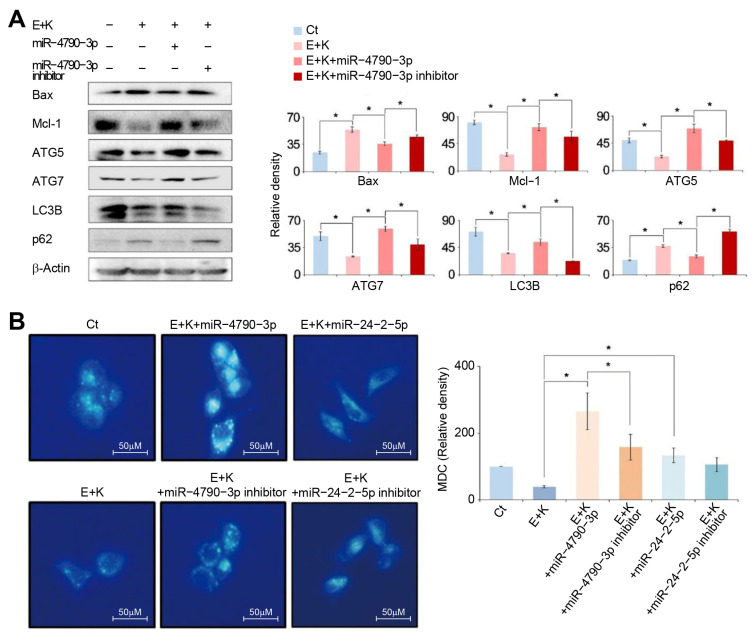
The miR-4790-3p inhibition test for the determination of the role of miR-4790-3p. (**A**) Western blot analysis in HepG2 cells with miR-4790-3p inhibition following individual treatments. Inhibition of miR-4790-3p led to upregulation of Bax, downregulation of Mcl-2, and antiapoptotic alterations (lower expression of ATG5, ATG7, and LC3B and higher expression of p62). (**B**) Determination of the degree of autophagy using immunofluorescences with MDC staining. The degree of autophagy was determined in HepG2 cells with up- or downregulation of miR-4790-3p and miR-24-2-5p, respectively, following individual treatments. While overexpressing miR-4790-3p promoted autophagy (bright blue), inhibiting miR-4790-3p led to reduced autophagy (darked blue). Overexpressing or inhibiting miR-24-2-5p led to similar results as for miR-4790-3p; however, the results were not as prominent as for miR-4790-3p. Values are presented as means ± standard deviations of three independent experiments. Note: * *p* < 0.05. Abbreviations: Bax, Bcl-2-like protein 4; E, everolimus; K, Ku0063794; Mcl-1, myeloid cell leukemia 1; MDC, monodansylcadaverine.

To further clarify the relationship between autophagy and miR-4790-3p, we investigated the changes in apoptosis- and autophagy-related markers in HepG2 cells when miR-4790-3p was inhibited (Figure 4A). The inhibition of miR-4790-3p potentiated proapoptotic and antiautophagic effects of combination therapy. In particular, after inhibiting miR-4790-3p, Bax increased, Mcl-1 decreased, and the changes in autophagy-related markers indicated antiautophagy (downregulation of ATG5, ATG7, and LC3B, and upregulation of p62). We then used monodansylcadaverine (MDC)—the autofluorescent drug—for the analysis of the machinery involved in the autophagic process at the molecular level (Figure 4B). In MDC staining, the addition of miR-4790-3p increased autophagy and the inhibition of miR-4790-3p reduced autophagy in combination therapy. The results for the addition or inhibition of miR-24-2-5p at the time of combination therapy were similar to the addition or inhibition of miR-4790-3p; however, the results were not as prominent as for miR-4790-3p. Similar results were also obtained in the experiments using Hep3B HCC cells (Figure S7, Supplementary Materials).

### 2.4. Determining the Expression of miR-4790-3p and ZNF225

**Figure 5:**
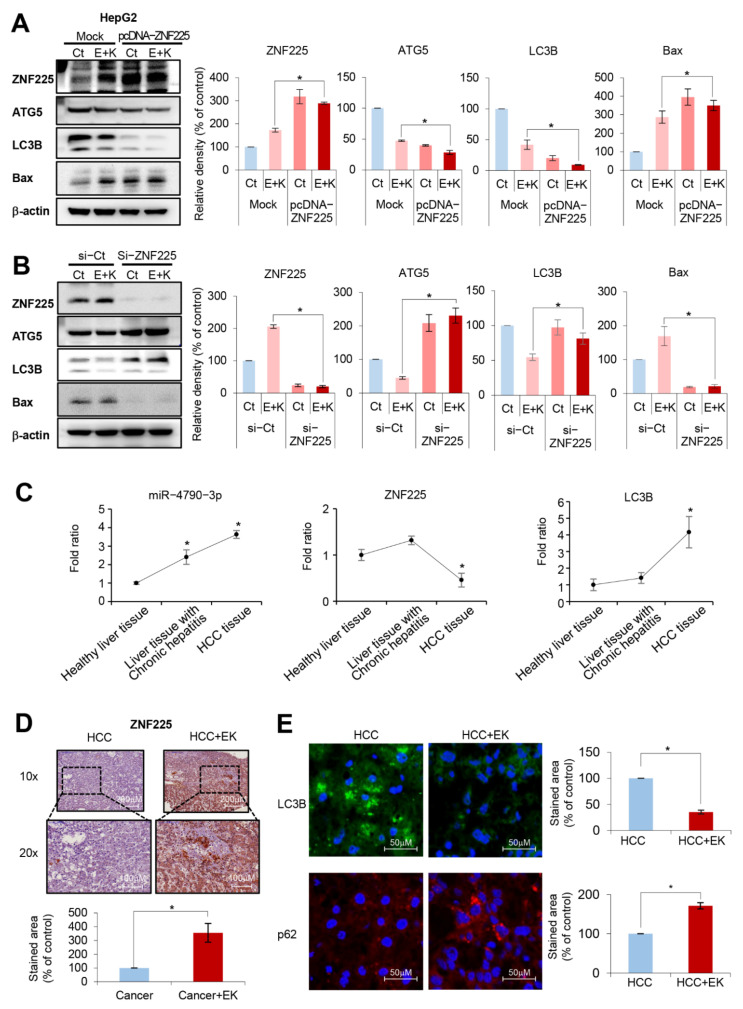
Determining the expression of miR-4790-3p and Zinc finger protein 225 (ZNF225) (**A**) Western blot analysis showing the effects of overexpressing ZNF225 on the expression of markers related to apoptosis and autophagy, respectively, in HepG2 cells. (**B**) Western blot analysis showing the effects of suppressing ZNF225 on the expression of markers related with apoptosis and autophagy, respectively, in HepG2 cells. (**C**) Comparison of miR-4790-3p and related markers between normal liver and HCC tissues. Real-time PCR indicated that the expression of miR-4790-3p was more significantly increased in the HCC tissues than in normal liver tissues (left). The expression of ZNF225 mRNA, a target mRNA of miR-4790-3p, was more significantly decreased in the HCC tissues than in normal liver tissues (middle). In addition, the expression of autophagy marker LC3B was significantly increased in the HCC tissues than in normal liver tissues (right). (**D**) ZNF225 immunohistochemistry showing the effect of everolimus and Ku0063794 combination therapy on the expression of ZNF225 in the ex vivo model of hepatocellular carcinoma (HCC). HCC tissues were cultured with 100 nM everolimus and 1 μM Ku0063794. After the combination therapy, the expression of ZNF225 was significantly increased in the HCC tissues. (**E**) Immunofluorescence showing the effect of everolimus and Ku0063794 combination therapy on the autophagy in the ex vivo model of hepatocellular carcinoma. The combination treatment led to downregulation of LC3B, as well as upregulation of p62, suggesting a decrease in autophagy. Values are presented as means ± standard deviations of three independent experiments. Note: * *p* < 0.05. Abbreviations: EK, everolimus plus Ku0063794 combination therapy; HCC, hepatocellular carcinoma.

To determine the role of ZNF225 in the HepG2 cells, a ZNF225 overexpression and suppression test was performed (Figure 5A,B). ZNF225 overexpression and suppression were achieved through transfection with pcDNA3.1Myc-ZNF225 and treatment with siZNF225, respectively. Western blot analysis showed that overexpressing ZNF225 led to reduced expression of proautophagic proteins (ATG5 and LC3B) and higher expression of a proapoptotic marker (Bax), which was reversed by the suppression of ZNF225(siZNF225). Similar results were obtained from the experiment using Hep3B cells (Figure S8). These results suggest that ZNF225 has a role in inhibiting autophagy and promoting apoptosis.

**Figure 6:**
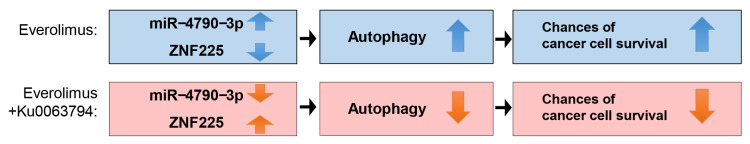
Possible mechanism of action comparing everolimus monotherapy and everolimus and Ku0063794 combination therapy.

We next examined the expression of miR-4790-3p in healthy liver tissue samples, the liver tissue samples with chronic hepatitis, and the HCC tissue samples of patients with HCC (Figure 5C). Real-time PCR indicated that the expression of miR-4790-3p was more significantly increased in the HCC tissues than in controls (*p* < 0.05). It was also found that the expression of ZNF225 mRNA, a target mRNA of miR-4790-3p, was more significantly decreased in the HCC tissues than in controls (*p* < 0.05). In addition, the expression of autophagy marker LC3B was more significantly increased in the HCC tissues than in controls (*p* < 0.05). Finally, we determined the effect of everolimus and Ku0063794 combination therapy on the expression of ZNF225 in the ex vivo model of HCC. HCC tissues were cultured with 100 nM everolimus and 1 μM Ku0063794. After the combination therapy for 48 h, it was found that the expression of ZNF225 was significantly increased in the HCC tissues (*p* < 0.05) (Figure 5D). Subsequently, we determined the effect of everolimus and Ku0063794 combination therapy on the autophagy in the ex vivo model of hepatocellular carcinoma. The combination treatment led to downregulation of LC3B as well as upregulation of p62, suggesting autophagy reduction (*p* < 0.05) (Figure 5E). Taken altogether, Figure 6 illustrates the possible mechanism of action, comparing everolimus monotherapy and everolimus and Ku-0063794 combination therapy. We think that combination therapy could be reasonably applied for patients with HCC because combination therapy has the potential to reduce autophagy by decreasing the expression of miR-4790-3p.

## 3. Discussion

Everolimus and Ku0063794 combination therapy significantly reduces viability, increases apoptosis, and reduces autophagy in HCC cells compared to individual monotherapies. In this study, we attempted to explain the superiority of combination therapy using the alternations of miRNAs according to the treatment modalities. The combination therapy led to significantly lower expression of miR-4790-3p and higher expression of ZNF225, respectively. ZNF225 is predicted to be the target of miR-4790-3p (http://www.targetscan.org. accessed date: 1 August 2015), and is closely related to FOSL2, JUNB, and JUND, all of which play essential roles in the autophagic processes. The increased expression of ZNF225 following combination therapy could be attributed to the decreased expression of miR-4790-3p, which has a role in suppressing the expression of ZNF225. The functional study of miR-4790-3p and ZNF225 revealed that regarding autophagy, miR-4790-3p promoted it, while ZNF225 inhibited it. In addition, regarding apoptosis, miR-4790-3p inhibited it, while ZNF225 promoted it. HCC tissues exhibited higher expression of miR-4790-3p and lower expression of ZNF225 compared to normal liver tissues; in addition, they exhibited higher expression of a proautophagic marker (LC3B). However, treatment of HCC tissues with the combination therapy led to higher expression of ZNF225; the combination therapy also led to downregulation of LC3B, as well as upregulation of p62, suggesting autophagy reduction. Taken together, our study suggests that the potentiated anticancer effect of the everolimus and Ku0063794 combination therapy is strongly associated with reduced autophagy resulting from diminished expression of miR-4790-3p, as well as higher expression of ZNF225.

In this study, we clarified the reason why the therapeutic effects of combination therapy showed great differences from each monotherapy in the context of miRNAs. The miRNAs are small, non-coding RNAs composed of about 22 nucleotides targeting mRNAs for cleavage and translational suppression [9]. The miRNAs bind to the 3′-untranslated region (3′-UTR) of target mRNAs after transcription, inhibiting the translation process, while exquisitely responding to the metabolic and stress conditions of the cells. Importantly, the mechanisms of action of miRNAs are affected by a variety of pharmaceutical compounds [10]. For instance, resveratrol downregulates the oncogenic miR-21, miR-30a-5p, and miR-19, thereby upregulating the targets of these miRNAs, resulting in the inhibition of glioma cell growth [11]. Curcumin upregulates miR-7 in pancreatic cancer cells, thereby downregulating lysine methyltransferase SET-8—the oncogenic target of miR-7—resulting in the inhibition of growth and invasion of pancreatic cancer cells [12]. Therefore, the mechanisms of various pharmaceutical compounds can be identified more clearly when they are related to the alterations of miRNAs by the compounds. Therefore, the mechanisms of various pharmaceutical compounds could be identified more clearly in the context of miRNAs.

Our study determined the roles of miR-4790-3p and ZNF225 in relation to autophagy. To date, little is known about the function of miR-4790-3p. Moustafa et al. identified miR-4790-3p as one of 118 upregulated miRNAs in prostate tumor cells [13]; in contrast, Wu et al. identified miR-4790-3p as one of 96 downregulated miRNAs in colorectal cancer tissues [14]. This study revealed that miR-4790-3p could increase autophagy by inhibiting the expression of the ZNF225. ZNF225 is a human protein that is encoded by the ZNF225 gene and is predicted to be the target of miR-4790-3p. It is one of the protein subtypes that contain the zinc finger—a small protein structural motif—that is characterized by the coordination of one or more zinc ions (Zn^2+^) in order to stabilize the fold. Visualization of potential network interactions of ZNF225 revealed that ZNF225 is closely related to FOSL2, JUNB, and JUND, all of which play essential roles in the autophagic processes. Data from previous in vitro studies consistently support the notion that zinc is critical for early and late autophagy [15,16,17,18]; of note, early and late autophagy were inhibited in cells treated with zinc chelators [19]. In healthy liver tissue, the expression levels of miR-4790-3p and ZNF225 were found to be depressed and elevated, while opposite results were found in HCC tissues. Furthermore, HCC tissues were characterized by the significantly higher expression of a proautophagic marker (LC3B). However, the treatment of HCC tissues with the combination therapy reversed this trend—the expression of ZNF225 was significantly increased and the expression of autophagic markers indicated autophagic reduction. In addition, the functional study regarding miR-4790-3p and ZNF225 indicated they are related to the promotion and reduction of autophagy, respectively.

In normal liver cells, autophagy prevents the development of malignant transformation by maintaining cell homeostasis [20,21,22]; however, once a tumor is established, autophagy could enhance the survival of HCC cells in the tumor microenvironment [20,23]. As the tumor advances, HCC cells are put into a metabolically demanding or challenging environment—limited blood supply, hypoxic environment, and marked inflammation—which necessitates autophagy in order to acquire energy for growth and survival [24]. It was revealed that autophagy is upregulated in up to 50–60% of tumor cells when they are cultured in a hypoxic environment [25,26,27,28]. Advanced HCC cells exhibited the increased autophagic flux [29,30], which also showed a positive correlation with tumor progression and poor prognosis of HCC [31]. Our study is consistent with previous publications, whereby HCC tissues showed higher autophagy than healthy liver tissues, and the treatment to reduce autophagy—including everolimus and Ku0063794 combination therapy—could increase apoptosis of HCC cells, because HCC cells essentially depend on autophagy for their survival. Therefore, this study suggests that the pharmacologic or biological blockage of autophagic flux could be one of the efficient anticancer strategies against HCCs.

The pharmaceutical compounds with antiautophagic properties include hydroxychloroquine, chloroquine, thapsigargin, azithromycin, 3-methyladenine, monensin, matrine, wortmannin, vorinostat, lucanthone, xanthohumol, and concanamycin A [32]. Of these, hydroxychloroquine and chloroquine have been approved by the FDA and have demonstrated their antitumor effects in various tumor models [33,34,35]. Recently, researchers have developed novel autophagy inhibitors that interfere with the binding between autophagosomes and lysosomes [36,37,38]. In addition, a number of studies have been conducted to determine the effectiveness of combining autophagy inhibitors with existing therapies, such as 5-fluorouracil, sorafenib, cisplatin, and oxaliplatin, in HCC models [39,40,41]. For instance, the co-administration of sorafenib and chloroquine has been reported to significantly reduce HCC growth rather than the monotherapy of either agent [40,42]. Currently, numerous clinical trials using autophagy inhibitors are underway, and thus it is expected to be possible to develop novel therapies interfering with autophagy in tumor cells in the near future.

## 4. Materials and Methods

### 4.1. Chemicals and Reagents

Everolimus and Ku-0063794 were obtained from Selleckem (Houston, TX, USA). Monodansylcadaverine (MDC) was purchased from Sigma-Aldrich (St. Louis, MO, USA). Lipofectamine RNAiMAX transfection reagent was purchased from Invitrogen (Carlsbad, CA, USA).

### 4.2. Cell Culture

Human hepatoblastoma cell line HepG2 and human hepatocellular carcinoma cell line Hep3B were obtained from Korean Cell Line Bank (KCLB, Seoul, Republic of Korea). HepG2 and Hep3B cells were maintained in DMEM–high-glucose medium (Thermo Fisher Scientific, Waltham, MA, USA). The medium was supplemented with 10% fetal bovine serum (FBS; GibcoBRL, Calsbad, CA, USA) and 1% penicillin and streptomycin (Thermo Fisher Scientific MA, USA) at 37 °C in saturating humidity with 5% CO_2_ in an incubator.

### 4.3. Cell Viability Assay

The cell viability assay was evaluated with water soluble tetrazolium salt (WST-1) using an EZ-Cytox Cell Viability Assay Kit (Itsbio, Seoul, Korea) according to the manufacturer’s instructions. Briefly, HepG2 and Hep3B cells were cultured in 96-well plates (1 × 10^4^ cells per well). The HepG2 and Hep3B cells were treated with everolimus and Ku-0063794 for 24 and 48 h, respectively. Then, the reagent from the EZ-Cytox Cell Viability Assay Kit was applied to each well. Absorbance was measured at 450 nm using the multi-mode reader (Bio-Tek, Winooski, VT, USA)

### 4.4. Western Blot Analysis

HepG2 and Hep3B cells were lysed using the EzRIPA Lysis Kit (ATTO Corporation, Tokyo, Japan) and quantified using Bradford reagent (Bio-Rad, Hercules, CA, USA). Proteins were visualized by Western blot analysis using the following primary antibodies (1:1000 dilution) at 4 °C overnight and then with HRP-conjugated secondary antibodies (1:2000 dilution) for 1 h at 25 °C. Primary antibodies against Bcl-2-like protein 4 (Bax), myeloid cell leukemia 1 (Mcl-1), ATG5, ATG7, LC3B, p62, and β-actin were obtained from Cell Signaling Technology (MA). Horseradish peroxidase (HRP)-conjugated secondary antibody were obtained from Vector Labs (Burlingame,WA, USA). Specific immune complexes were detected using the Western Blotting Plus Chemiluminescence Reagent (Millipore, Bedford, MA, USA).

### 4.5. Monodansylcadaverine (MDC) Staining

HepG2 and Hep3B cells were cultured on Lab-Tek chamber slides (Thermo Fisher Scientific, Hemel Hempstead, UK) and the HepG2 and Hep3B cells were treated with everolimus and Ku-0063794 for 24 and 48 h, respectively. Subsequently, HepG2 cells were stained with 0.05 mM MDC at 37 °C for 30 min. The autophagic vacuoles were observed using a laser scanning microscope (Eclipse TE300; Nikon, Tokyo, Japan).

### 4.6. Validation of miRNA Expression in Human HCC Tissues

Real-time PCR analysis was performed to validate miRNA expression in human HCC tissues. This study was approved by the institutional review board at Daejeon St. Mary’s Hospital, the Catholic University of Korea. Total RNA was extracted using the mirVana^TM^ miRNA isolation kit according to the manufacturer’s protocol. Reverse transcription was performed with 1 μg RNA using a universal cDNA synthesis kit (Exiqon, Vedbaek, Denmark). The primers used for ExiLENT SYBR Green master mix (Exiqon, Vedbaek, Denmark) were the hsa-miR-24-2-5p and hsa-miR-4790-3p primer sets (Exiqon, Vedbae, Denmark). The reaction was performed using an Applied Biosystems 7500 96-well real-time PCR system (Life Technologies, Carlsbad, CA, USA). After normalization to U6 snRNA, the expression levels for each target gene were calculated using the comparative threshold cycle method.

### 4.7. miRNA Transfection

The miR-4790-3p and miR-24-2-5p mirVana miRNA mimics were purchased from Ambion, Inc. (Bedford, MA, USA). Transfection was carried out using RNAiMAX (Invitrogen, Carlsbad, CA, USA), according to the manufacturer’s instructions.

### 4.8. Microarray

Human microRNA expression was analyzed with miRCURY LNA^TM^ microRNA Array (Exiqon, Vedbaek, Denmark), covering 1918 well-characterized human microRNAs among 3100 capture probes for human, mouse, and rat miRNAs. In this procedure, 5′-phosphate from 250 ng of total RNA was removed by treating with Calf Intestinal Alkaline Phosphatase (CIP) followed by labeling with Hy3 green fluorescent dye. Labeled samples were subsequently hybridized by loading onto a microarray slide using a Hybridization Chamber Kit part #G2534A (Agilent Technologies, Santa Clara, CA, USA) and Hybridization Gasket Slide Kit part #G2534-60003 (Agilent Technologies). Hybridization was performed over 16 h at 56 °C, followed by washing of the microarray slide as recommended by the manufacturer. Processed microarray slides were then scanned with an Agilent G2565CA Microarray Scanner System (Agilent Technologies, Santa Clara, CA, USA). Scanned images were imported using Agilent Feature Extraction software version 10.7.3.1 (Agilent Technologies, Santa Clara, CA, USA) and the fluorescence intensities of each image were quantified using the modified Exiqon (Vedbaek, Denmark) protocol and corresponding GAL files.

### 4.9. Overexpression and Silencing of the ZNF Genes

Human ZNF cDNA were obtained by RT-PCR using HepG2 cell RNA. PCR products were digested with *HindIII* and *Xho*I (Takara Tokyo, Japan) and ligated into the pcDNA3.1 Myc-His vector (Invitrogen, Carlsbad, CA, USA). Transcription was specifically suppressed by the introduction of the 21-nucleotide duplex siRNA, which targeted the zinc finger (ZNF) mRNA coding sequence. Briefly, HepG2 and Hep3B cells were plated in 6-well plates (2 × 10^5^ cells/well) and transiently transfected with 100 nM per well of ZNF siRNA (Santa Cruz, Santa cruz, CA, USA) mixed with the Lipofectamine transfection reagent (Thermo Fisher Scientific, Waltham, MA, USA) according to the manufacturer’s instructions. After 5 h incubation, the medium was changed to complete culture medium and the cells were incubated at 37 °C in a CO_2_ incubator for 48 h before harvesting.

## 5. Quantification of Apoptosis by Flow Cytometry

To determine the proportion of cells undergoing apoptotic cell death, HepG2 and Hep3B cells were stained with Annexin V/propidium iodide (PI) (BD Biosciences, Flanklin Lakes, NJ, USA). After incubation for 10 min in the dark at 25 °C, the cells were analyzed using an Attune NxT Acoustic focusing cytometer (Thermo Fisher Scientific, Carlsbad, CA, USA).

### 5.1. TUNEL Assay

TUNEL analysis was performed for the detection of apoptosis in HCC tissues using the in situ Apoptosis Detection Kit (Takara, Tokyo, Japan) following the manufacturer’s instructions. In brief, sample slides were incubated with 50 μL of TUNEL reaction mixture and TdT labeling reaction mix for 1 h at 37 °C in the dark. After being rinsed with PBS three times, the samples were observed using a fluorescence imaging system (EVOS U5000; Invitrogen, Carlsbad, CA, USA).

### 5.2. Ex Vivo Culture of Hepatocellular Carcinoma Tissue

Human hepatocellular carcinoma tissue specimens (paired normal and cancer tissues from each patient, *n* = 10) were obtained after surgical resections performed at our institution. The ethics committee at our institution approved the use of the tissue specimens for the research.

Human HCC tissues were cultured in DMEM/F12 media supplemented with 10% fetal bovine serum (FBS; GibcoBRL, Calsbad, CA, USA) and 1% penicillin and streptomycin (Thermo, Waltham, MA, USA) at 37 °C in saturating humidity with 5% CO_2_ in an incubator. The tissues were treated with 100 nM everolimus and 1 μM Ku0063794 or vehicle solution. After the treatment for 24 h, the tissues were processed for histological analyses.

### 5.3. Immunohistochemical Analyses

For immunohistochemical analyses, formalin-fixed, paraffin-embedded (FFPE) tissue sections were deparaffinized, rehydrated in an ethanol series, and subjected to epitope retrieval using standard procedures. Primary antibodies to ZNF225, LC3B, p62, and cleaved caspase-3 were used for immunochemical stains. ZNF225, LC3B, p62, and cleaved caspase-3 were obtained from Cell Signaling Technology (MA). Horseradish peroxidase (HRP)-conjugated secondary antibody was obtained from Vector Labs (CA). LC3B and p62 were observed using a fluorescence imaging system (EVOS U5000; Invitrogen, CA, USA). ZNF225 was observed using a panoramic distal slide scanner system (3D HISTECH; Budapest, Hungary).

### 5.4. Statistical Analysis

All data were analyzed using SPSS 11.0 software (SPSS Inc.; Chicago, IL, USA) and are presented as the means ± SDs. Statistical comparisons between the mean values of two groups were performed using Mann–Whitney U tests; to compare three or more groups, Kruskal–Wallis tests were used. Probability (*p*) values of <0.05 were considered statistically significant.

## 6. Conclusions

The combination therapy of everolimus and Ku0063794 significantly reduced viability, increased apoptosis, and reduced autophagy in HCC cells compared to individual monotherapies. We found that the superiority of the combination therapy is closely related to the alteration of miRNAs according to the treatment modalities. The combination therapy led to the significant decreased expression of miR-4790-3p and the increased expression of ZNF225—the predicted target of miR-4790-3p. In addition, the functional study regarding miR-4790-3p and ZNF225 indicated that they are related to the promotion and reduction of autophagy, respectively. It was also revealed that HCC tissues are characterized by higher expression of miR-4790-3p and lower expression of ZNF225; they are also characterized by higher autophagic flux. We, thus, conclude that the potentiated anticancer effect of the everolimus and Ku0063794 combination therapy is strongly associated with reduced autophagy resulting from diminished expression of miR-4790-3p, as well as higher expression of ZNF225.

## Data Availability

The data presented in this study are openly available.

## Supplementary Materials

The Supplementary Materials are available online at https://www.mdpi.com/1422-0067/22/6/2859/s1.

## Abbreviations

Bax	Bcl-2-like protein 4
E	Everolimus
K	Ku0063794
Mcl-1	Myeloid cell leukemia 1
miR-NC	miRNA mimic negative control
MDC	Monodansylcadaverine
EK	Everolimus plus Ku0063794 combination therapy
HCC	Hepatocellular carcinoma
