# Supplementary material for: Combining Everolimus and Ku0063794 Promotes Apoptosis of Hepatocellular Carcinoma Cells via Reduced Autophagy Resulting from Diminished Expression of miR-4790-3p
Source: Int J Mol Sci. 2021 Mar 11;22(6):2859. doi: 10.3390/ijms22062859 (PMC7998287; doi:10.3390/ijms22062859)
Supplement: Supplementary file 1 [file ijms-22-02859-s001.zip › ijms-1056100-supplementary.docx]

**< Supplementary Figure legends>**

**Supplementary Figure 1.** Quantitative analysis of apoptosis using Annexin V/PI staining and flow cytometry following each treatment in HepG2 and Hep3B cells, respectively. Apoptotic cell proportion is expressed as the total percentage of Annexin V/PI-positive cells.

**Supplementary Figure 2.** Western blot analysis comparing the expression of LC3B in the absence/presence of autophagy inhibitor bafilomycin A1 in HepG2 and Hep3B cells, respectively. Autophagic flux was thus pharmacologically blocked using bafilomycin A1, which prevents maturation of autophagic vacuoles by inhibiting fusion between autophagosomes and lysosomes. In the combination therapy group, significant reduction of the expression of LC3B was observed following the treatment of bafilomycin A1, suggesting reduced autophagy.

**Supplementary Figure 3.** Comparison of expressions of miRNAs in mono- and combination therapies of everolimus and Ku0063794 in Hep3B cells.

(A) Western blot analysis showing the effect of each treatment on the expression of p-mTOR in Hep3B cells.

(B) Cell viability assay of Hep3B cells following mono- and combination therapies of everolimus and Ku0063794. Compared to the monotherapies of everolimus and Ku0063794, the combination therapy significantly reduced the viability of Hep3B cells.

(C) Western blot analysis of Hep3B cells following mono- or combination therapies. Combination therapy induced a significant increase in the expression of Bax, a decrease in the expression of Mcl-1, and a decrease in the expression of autophagy markers.

Values are presented as mean ± standard deviation of three independent experiments. * P < 0.05. Abbreviations: Bax, Bcl-2-like protein 4; E, everolimus, K, Ku0063794, Mcl-1, myeloid cell leukemia 1.

**Supplementary Figure 4.** The expression profiles of miRNAs in HepG2 cells following treatments with everolimus or Ku0063794 alone or in combination.

**Supplementary Figure 5.** Changes of miR-4790-3p and miR-24-2-5p in the Hep3B cells after everolimus and Ku0063794 mono- and combination therapies.

(A-B) Real-time PCR showing the expression of miR-4790-3p and miR-24-2-5p in Hep3B cells after individual treatments. Compared to the control group, the expression of miR-4790-3p and miR-24-2-5p was increased after everolimus monotherapy, slightly decreased after Ku0063794 monotherapy, and significantly decreased after the combination therapy.

(C) Real-time PCR showing the expression of ZNF225 and LC3B after the combination therapy. ZNF225 is known to be the target mRNA of miR-4790-3p. The mRNA expression of ZNF225 and LC3B was significantly up- and downregulated following combination therapy, respectively.

**Supplementary Figure 6.** Overexpression test for the determination of the role of miR-4790-3p and miR-24-2-5p in Hep3B cells.

(A) [Left] Real-time PCR analysis demonstrating higher miR-4790-3p expression in miR-4790-3p-transfected Hep3B cells 24 h after transfection. [Right] Western blot analysis in Hep3B cells with miR-4790-3p overexpression following individual treatments. Overexpression of miR-4790-3p led to downregulation of Bax, upregulation of Mcl-2, and pro-apoptotic alterations (higher expression of ATG5 and ATG7 and lower expression of p62).

(B) [Left] Real-time PCR analysis demonstrating higher miR-24-2-5p expression in miR-24-2-5p-transfected Hep3B cells 24 h after transfection. [Right] Western blot analysis in Hep3B cells with miR-24-2-5p overexpression following individual treatments. Western blot analysis following overexpressing miR-24-2-5p in Hep3B cells following individual treatments.

Overexpression of miR-24-2-5p did not lead to consistent alterations in the expression of pro-apoptotic, anti-apoptotic, and autophagic proteins.

Values are presented as mean ± standard deviation of three independent experiments. * P < 0.05. Abbreviations: Bax, Bcl-2-like protein 4; E, everolimus; K, Ku0063794; Mcl-1, myeloid cell leukemia 1; miR-NC, miRNA mimic negative control.

**Supplementary Figure 7.** Inhibition test for the determination of the role of miR-4790-3p.

(A) Western blot analysis in Hep3B cells with miR-4790-3p inhibition following individual treatments. Inhibition of miR-4790-3p led to upregulation of Bax, downregulation of Mcl-2, and anti-apoptotic alterations (lower expression of ATG5, ATG7, and LC3B and higher expression of p62).

(B) Determination of the degree of autophagy using immunofluorescences with MDC staining. The degree of autophagy was determined in Hep3B cells with up- or down-regulation of miR-4790-3p and miR-24-2-5p, respectively, following individual treatments. While overexpressing miR-4790-3p led to promoted autophagy, inhibiting miR-4790-3p led to reduced autophagy. Overexpressing or inhibiting miR-24-2-5p led to the similar results as miR-4790-3p; however, it was not as prominent as the miR-4790-3p cases.

Values are presented as mean ± standard deviation of three independent experiments. * P < 0.05. Abbreviations: Bax, Bcl-2-like protein 4; E, everolimus; K, Ku0063794; Mcl-1, myeloid cell leukemia 1, and MDC, monodansylcadaverine.

**Supplementary Figure 8.** Overexpression/suppression tests of ZNF225 for the determination of its role in Hep3B cells.

(A) Western blot analysis showing the effects of overexpressing ZNF225 on the expression of markers related with apoptosis and autophagy, respectively, in Hep3B cells.

(B) Western blot analysis showing the effects of suppressing ZNF225 on the expression of markers related with apoptosis and autophagy, respectively, in Hep3B cells
