# Supplementary figures and images for: Combining Everolimus and Ku0063794 Promotes Apoptosis of Hepatocellular Carcinoma Cells via Reduced Autophagy Resulting from Diminished Expression of miR-4790-3p
Source: Int J Mol Sci. 2021 Mar 11;22(6):2859. doi: 10.3390/ijms22062859 (PMC7998287; doi:10.3390/ijms22062859)

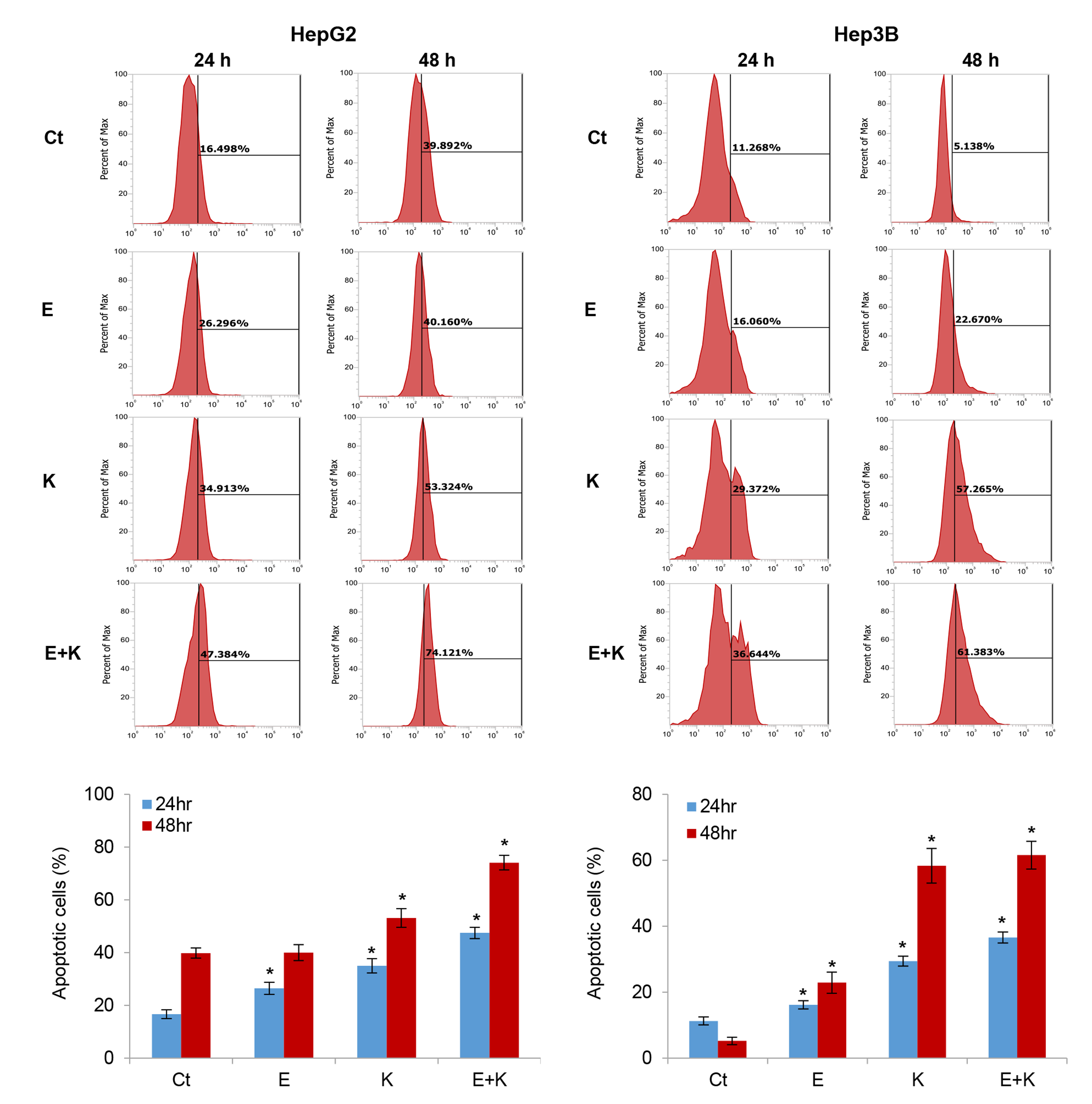

Supplement: Supplementary file 1 [file ijms-22-02859-s001.zip › Figure S1.tif]

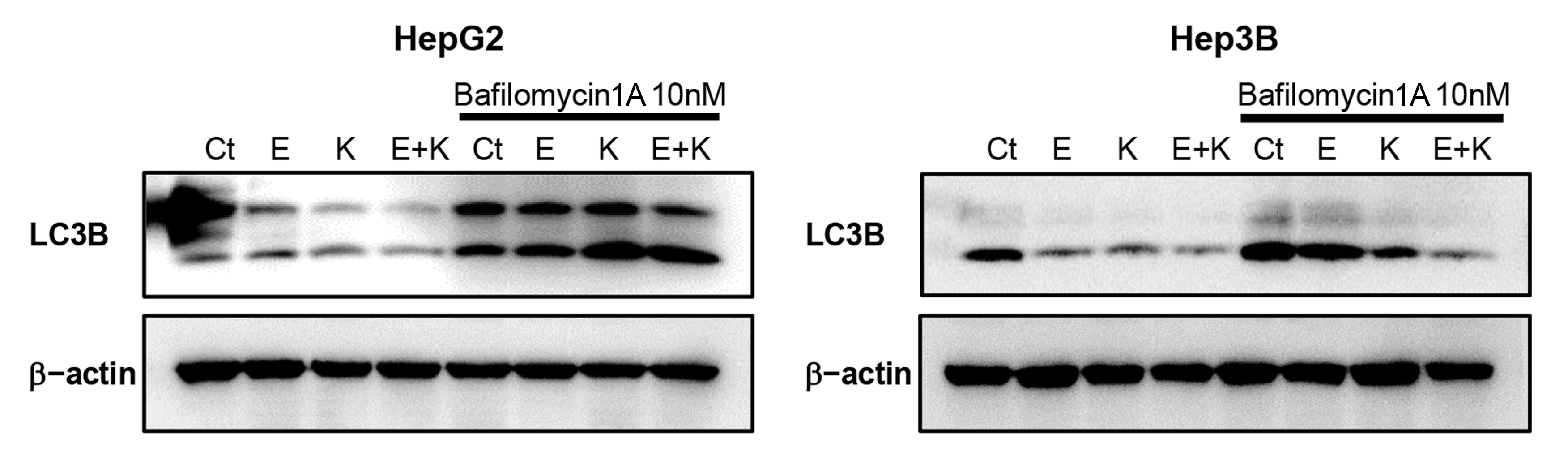

Supplement: Supplementary file 1 [file ijms-22-02859-s001.zip › Figure S2.tif]

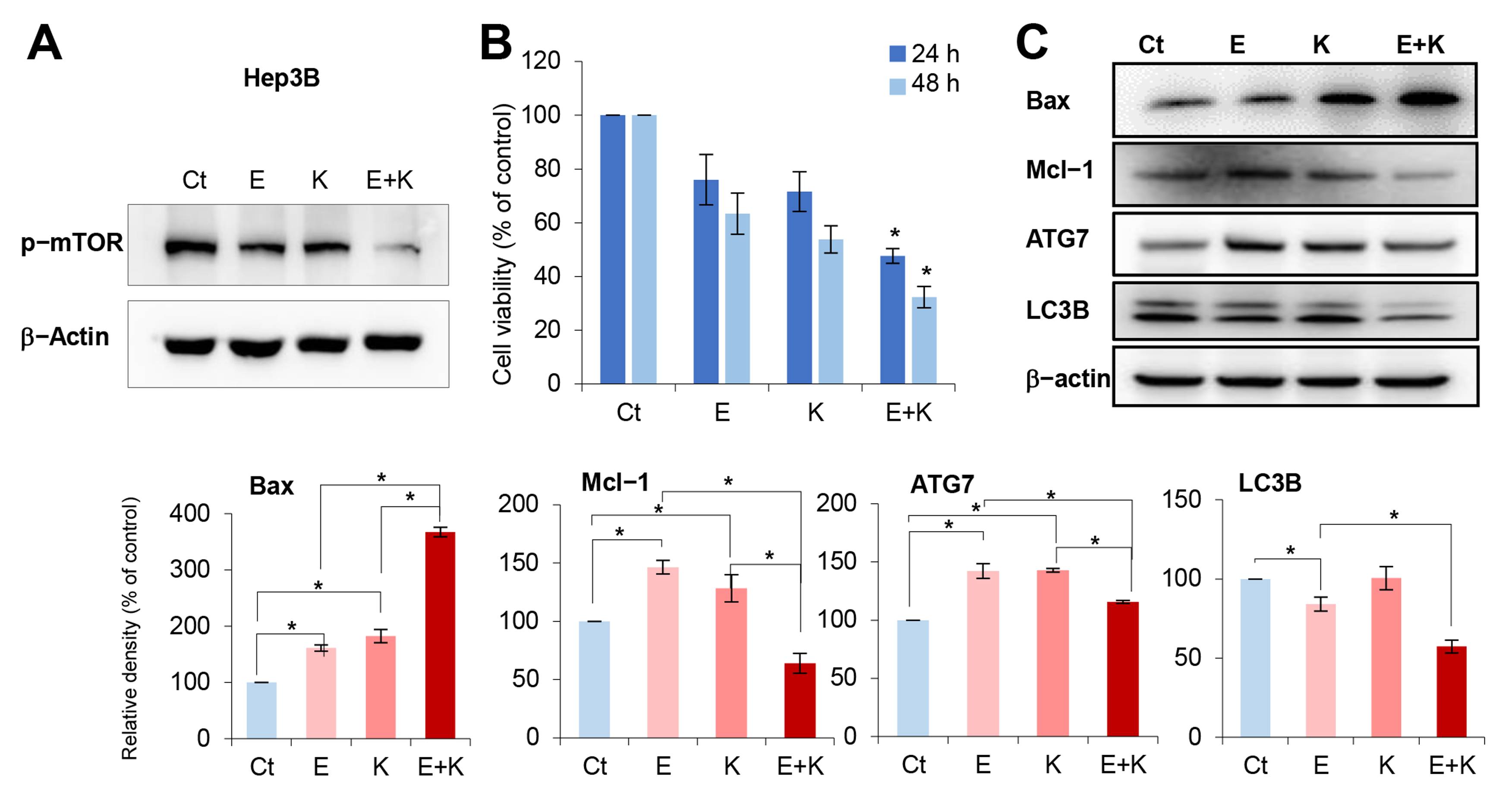

Supplement: Supplementary file 1 [file ijms-22-02859-s001.zip › Figure S3.tif]

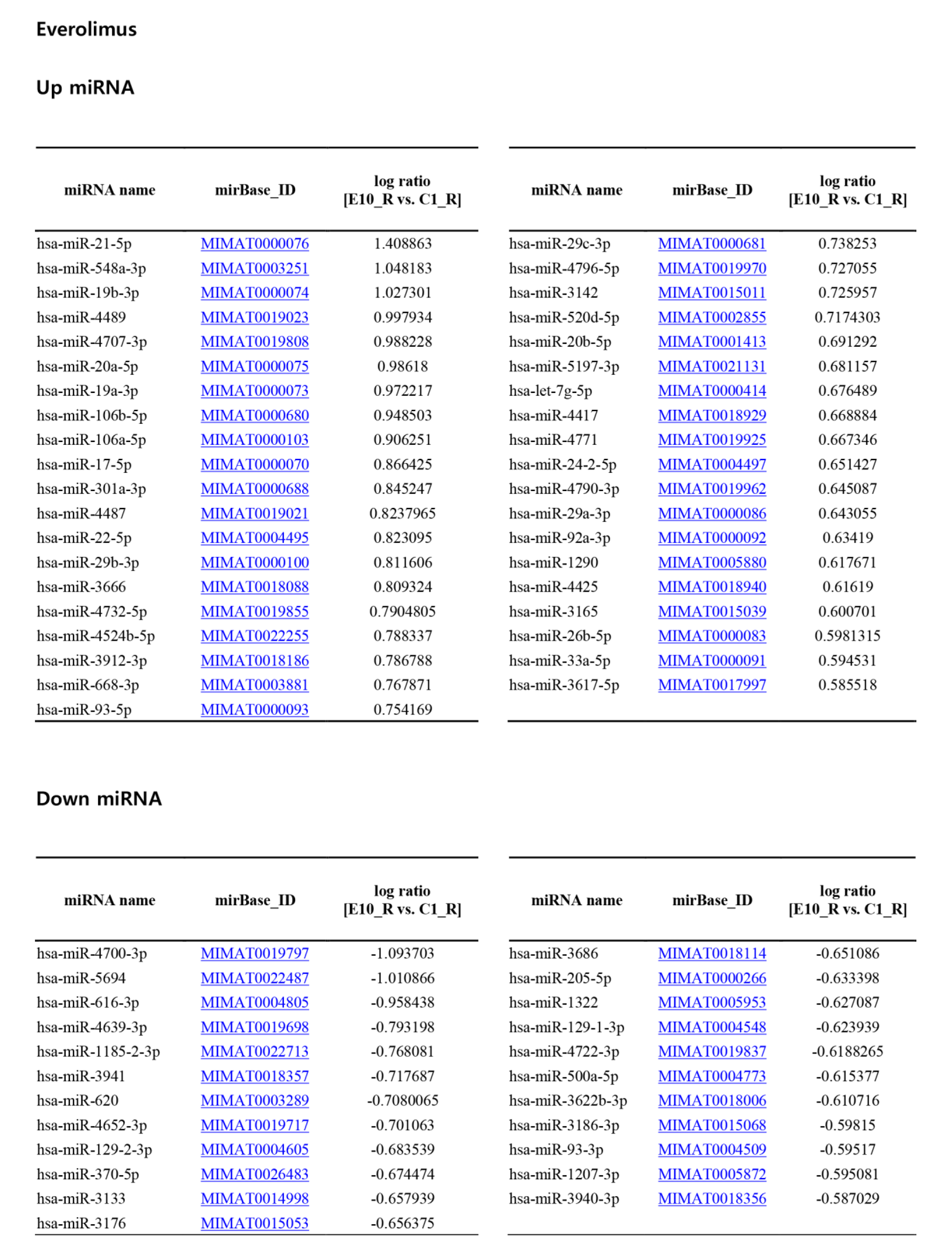

Supplement: Supplementary file 1 [file ijms-22-02859-s001.zip › Figure S4-1.tif]

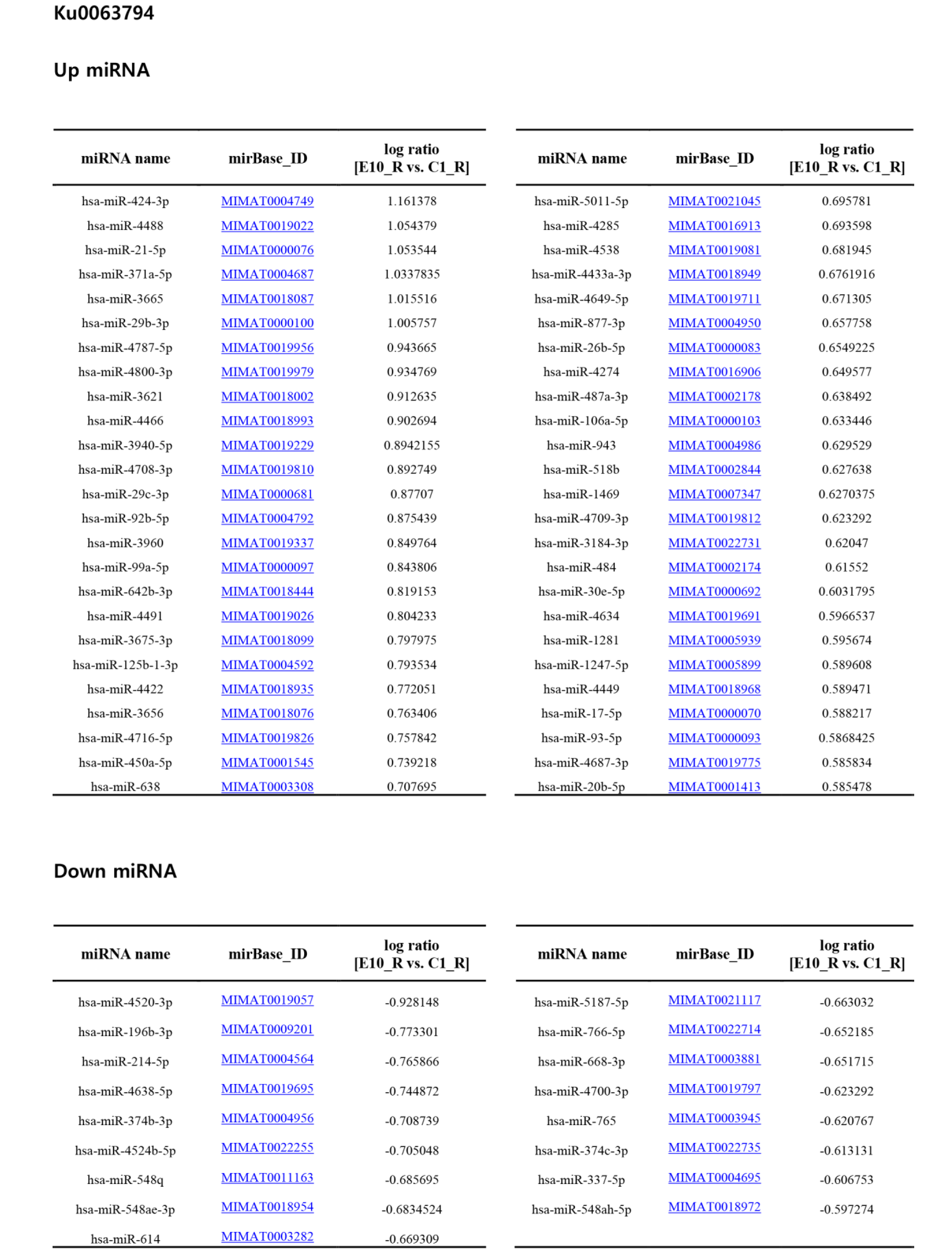

Supplement: Supplementary file 1 [file ijms-22-02859-s001.zip › Figure S4-2.tif]

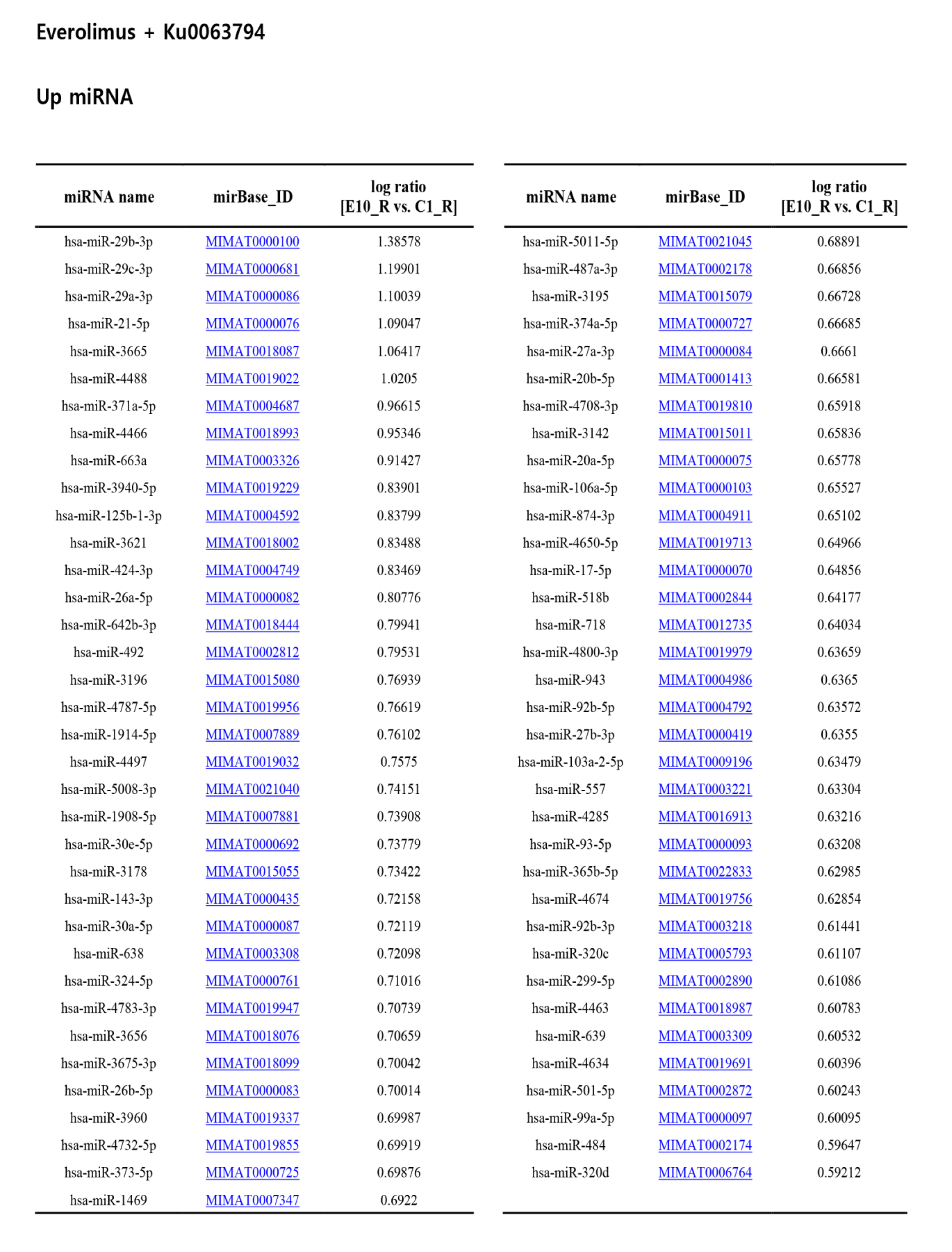

Supplement: Supplementary file 1 [file ijms-22-02859-s001.zip › Figure S4-3.tif]

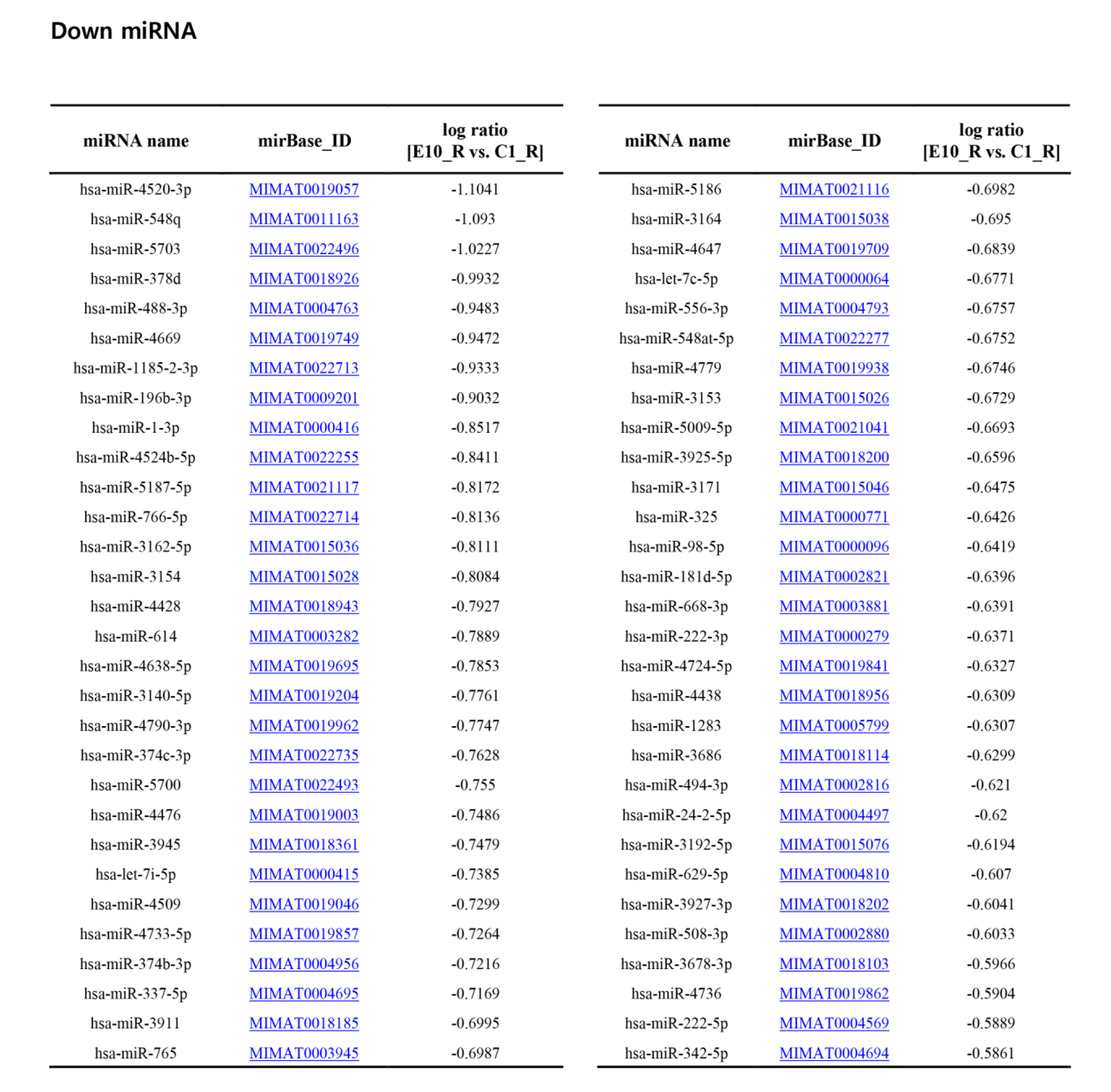

Supplement: Supplementary file 1 [file ijms-22-02859-s001.zip › Figure S4-4.tif]

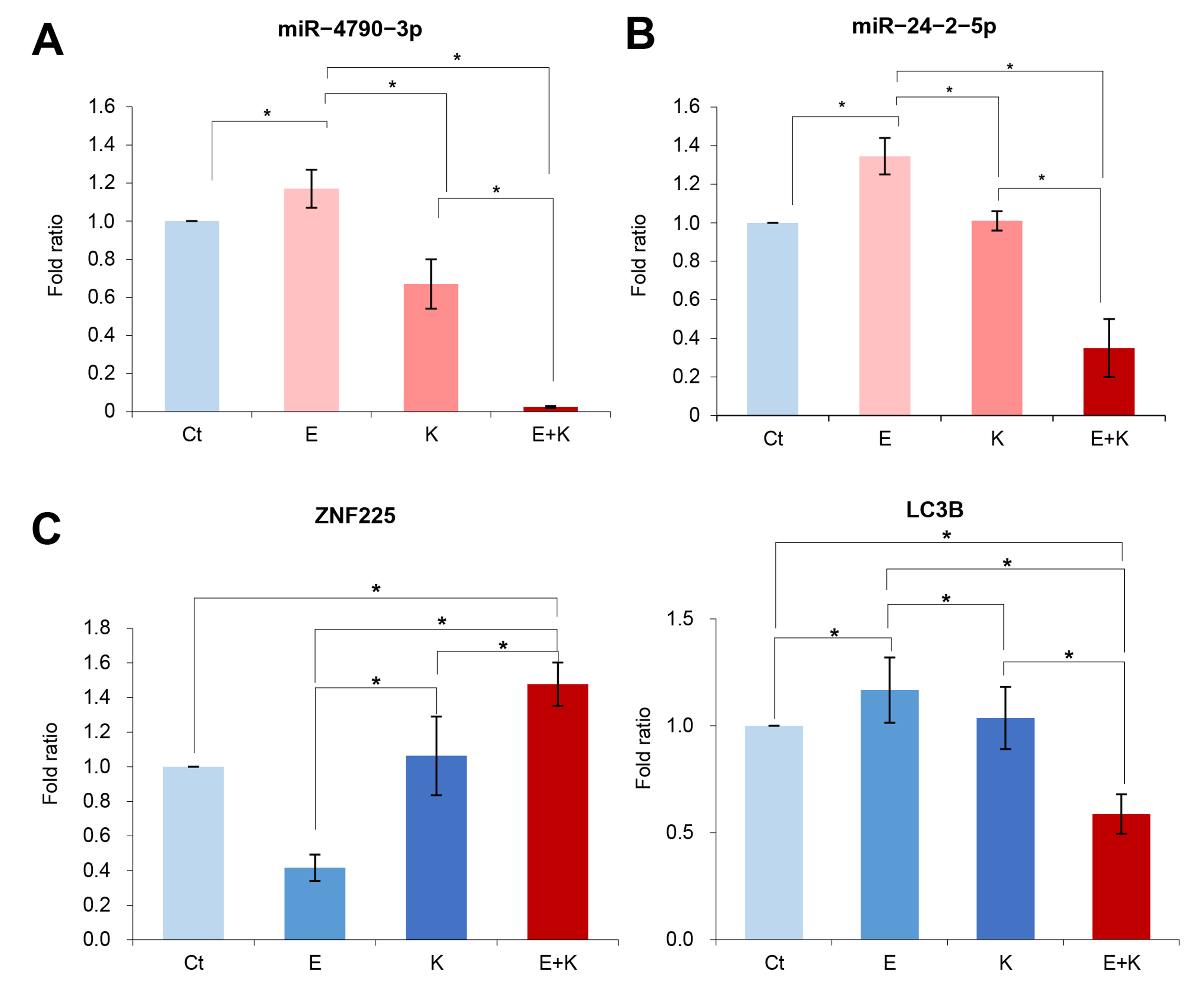

Supplement: Supplementary file 1 [file ijms-22-02859-s001.zip › Figure S5.tif]

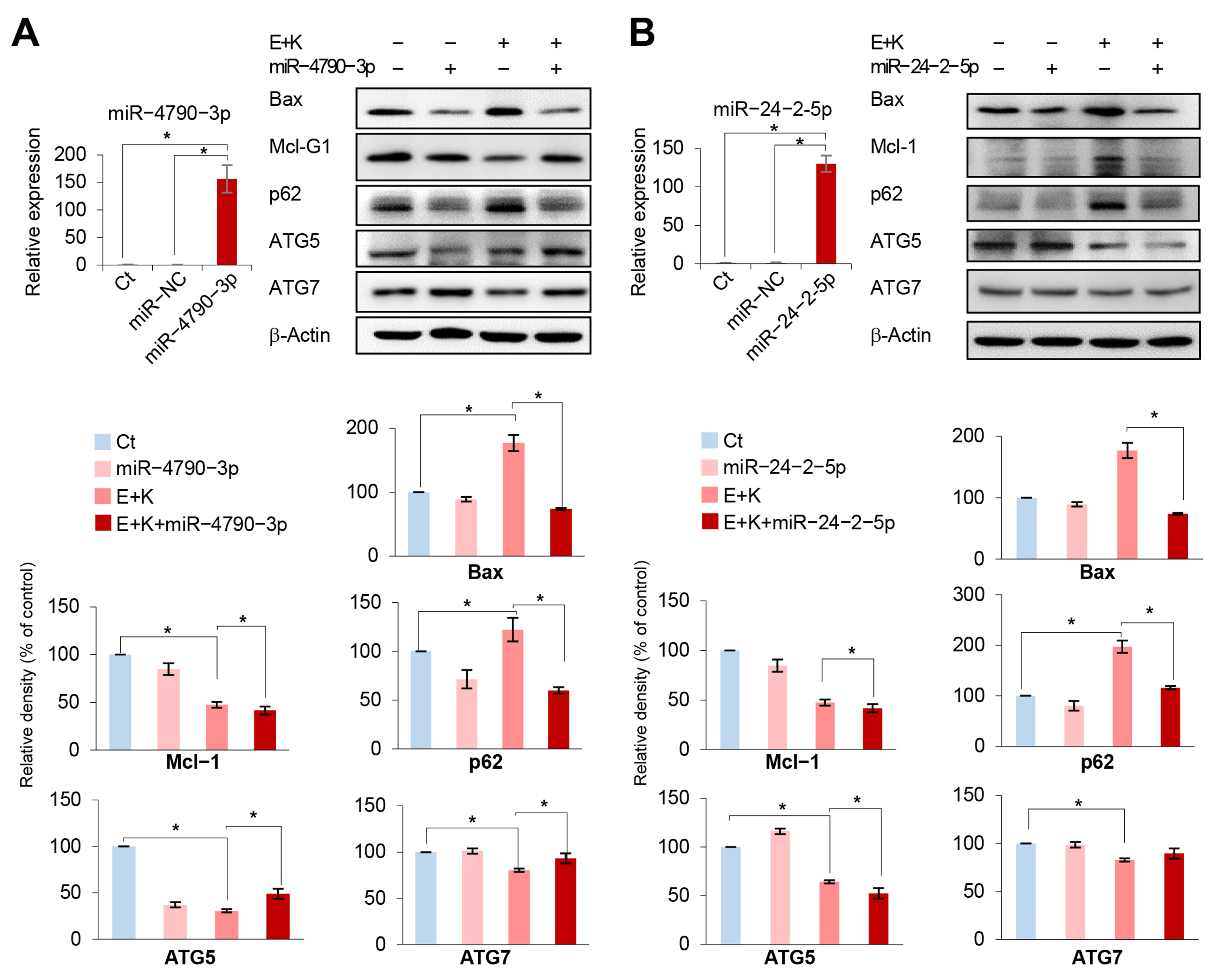

Supplement: Supplementary file 1 [file ijms-22-02859-s001.zip › Figure S6.tif]

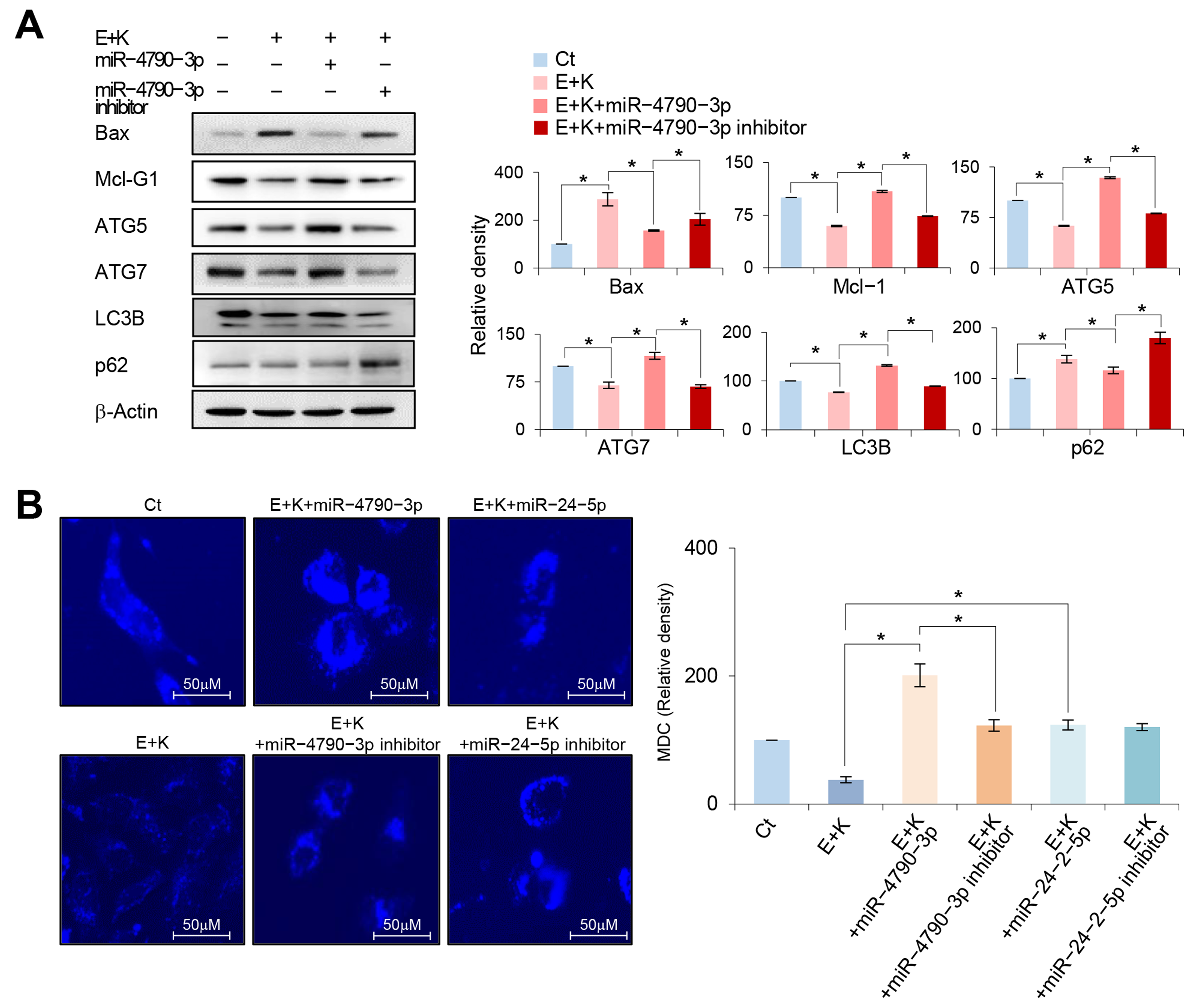

Supplement: Supplementary file 1 [file ijms-22-02859-s001.zip › Figure S7.tif]

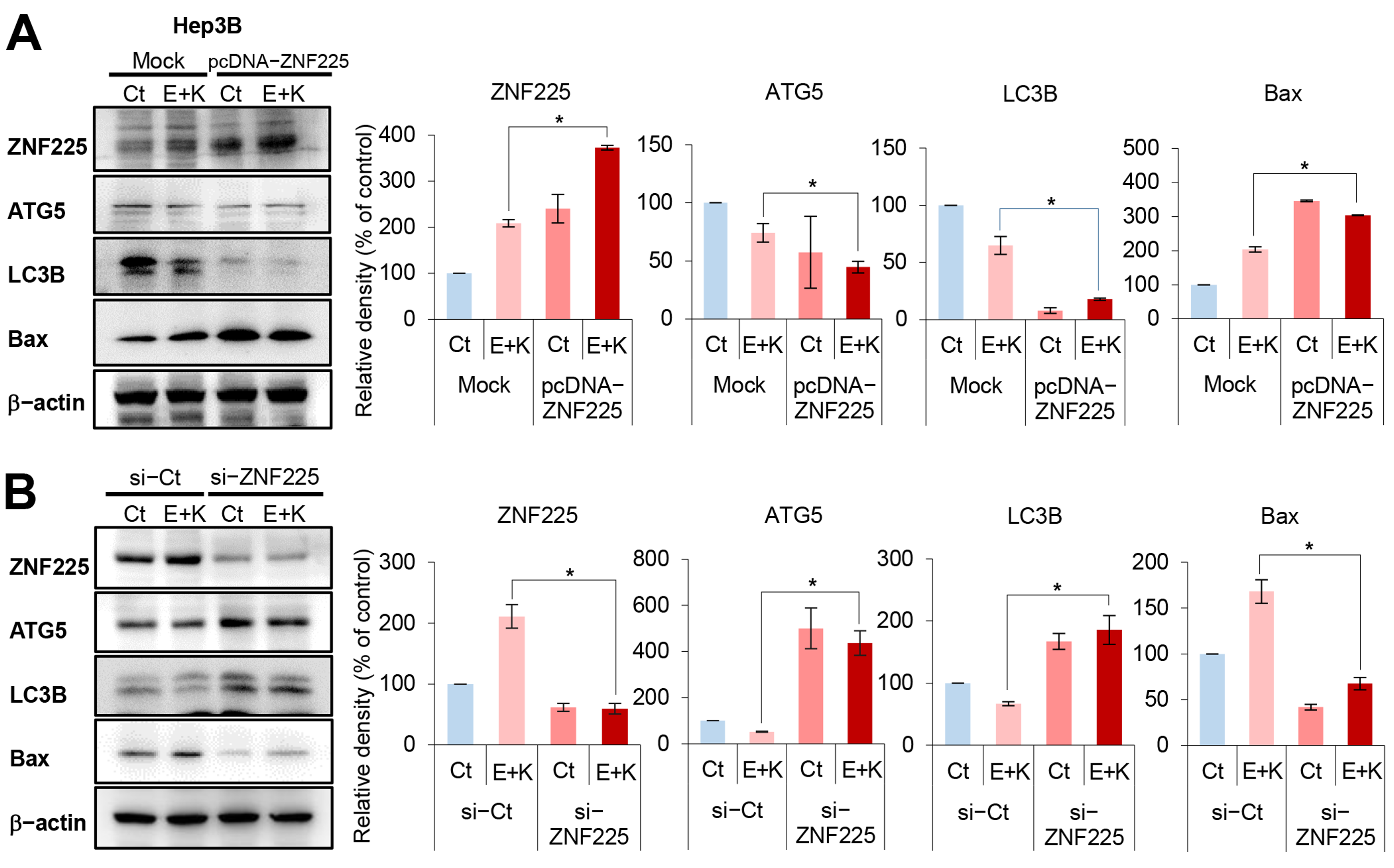

Supplement: Supplementary file 1 [file ijms-22-02859-s001.zip › Figure S8.tif]
